# Pyoderma Gangrenosum Post-Breast Surgery: A Case Report and Comprehensive Review of Management Strategies

**DOI:** 10.3390/jcm13133800

**Published:** 2024-06-28

**Authors:** Ioan Constantin Pop, Radu Alexandru Ilies, Corina Baican, Stefan Strilciuc, Valentin Muntean, Maximilian Muntean

**Affiliations:** 1 Plastic Surgery Department, “Prof. Dr. I Chiricuta” Institute of Oncology, 400015 Cluj-Napoca, Romania; drp.ionut@gmail.com (I.C.P.); iliesradu.14@gmail.com (R.A.I.); maximilian.muntean@iocn.ro (M.M.); 2 Dermatology Department, County Emergency Hospital, 400006 Cluj-Napoca, Romania; cibaican@yahoo.com; 3 Research Center for Functional Genomics, Biomedicine and Translational Medicine, Iuliu Hatieganu University of Medicine and Pharmacy, 400012 Cluj-Napoca, Romania; 4 RoNeuro Institute for Neurological Research and Diagnostic, 400337 Cluj-Napoca, Romania; 5 General Surgery Department, Humanitas Clinical Hospital, 400012 Cluj-Napoca, Romania; valentin.muntean@gmail.com

**Keywords:** pyoderma gangrenosum, breast surgery, management strategies, autoimmune skin conditions

## Abstract

**Background/Objectives**: Pyoderma gangrenosum (PG) is a rare, autoimmune skin condition characterized by painful, rapidly progressing ulcers, often associated with autoimmune dysregulation. Managing PG following breast surgery presents unique challenges due to its pathergy phenomenon, which complicates surgical interventions. This article outlines the case of PG in a 48-year-old female post-breast surgery and reviews management strategies through a systematic analysis of the literature. **Methods:** A systematic literature review from 2018 to 2023 identified 24 relevant articles on PG management post-breast surgery. The studies were analyzed to compare the efficacy and complications of conservative versus combined (conservative and surgical) treatment strategies. **Results:** Results indicate that while conservative management, primarily with corticosteroids, remains preferred, combined strategies, including systemic therapies, vacuum-assisted closure, and surgery, offer significant benefits in select cases. **Conclusions:** Our findings suggest that a personalized, multifaceted treatment plan is crucial for managing PG effectively, emphasizing the need for early detection, meticulous planning, and comprehensive care to optimize patient outcomes.

## 1. Introduction

Pyoderma gangrenosum (PG) is a rare, non-infectious dermatosis characterized by the rapid development of painful ulcers. Despite its unknown etiology, PG is recognized as an autoimmune disorder classified among neutrophilic dermatoses [1]. Managing PG is particularly challenging due to its rapid symptom progression, transforming from a pustule or inflammatory nodule into painful, ulcerative lesions with distinctive violaceous borders within a short time. Without appropriate management, PG can result in severe morbidity and long-term disability, highlighting the critical need for early diagnosis and treatment [2]. Additionally, a review suggests that surgical intervention should be reserved for severe cases and only after attempts to control the condition with medications [3]. PG is a complex disease with a multifactorial etiology, including genetic and environmental factors. Trauma, the most well-documented trigger, can initiate PG ulcerations and stimulate immunological dysregulation, particularly an aberrant response from innate and adaptive immune systems [4]. Studies suggest that aberrant innate immune system activation plays a key role in PG development. While most research focuses on the innate immune response and autoinflammation, alterations in the adaptive immune response are also involved. Dysregulated T-cell function, particularly an imbalance between Th1 and Th2 cells, is thought to play a role in the pathogenesis of the disease. Increased Th1 cell activity produces pro-inflammatory cytokines, while reduced Th2 cell activity affects the resolution of inflammation and wound healing. Additionally, elevated levels of regulatory T cells (Tregs) have been observed in patients with PG, potentially indicating impaired immune tolerance mechanisms [5]. Environmental triggers like trauma and infections can further exacerbate the disease in susceptible individuals. Understanding these triggers and their impact on the immune system is crucial for developing preventive measures and early interventions to reduce PG incidence and severity [4].

### 1.1. Clinical Presentation

PG is characterized by a distinctive clinical presentation, beginning typically as tender, erythematous papules or pustules. These initial lesions rapidly progress to form deep, necrotic ulcers that are distinguished for their irregular, undermined borders and a characteristic violaceous or dusky-red coloration. The size of these lesions can be significant, often extending through the dermis and occasionally involving deeper structures, such as tendons or bones. However, PG ulcers are susceptible to spread, often leading to the formation of satellite ulcers around the primary wound site. The evolution can vary from indolent to aggressive forms, with a peak incidence between 20 and 50 years of age [6].

### 1.2. Diagnosis

Timely and accurate diagnosis of PG is crucial for appropriate management and differentiation from other similar conditions. PG is currently diagnosed by exclusion, as there are no specific diagnostic criteria for the disease. Despite this, efforts such as those by Su et al. [7] have contributed to proposing a structured approach to diagnosis, highlighting both major and minor criteria. Major criteria include the presence of painful, necrolytic, rapidly progressing cutaneous ulcers with irregular violaceous borders and the necessity to rule out other causes of cutaneous ulceration. Minor criteria encompass a history suggestive of pathergy or the observation of cribriform scarring, associated systemic diseases, compatible histopathological findings, and a positive response to PG-specific treatments. In addition to these criteria, laboratory investigations play a crucial role in the diagnostic process. Tests such as a complete blood count, inflammatory markers (such as C-reactive protein and erythrocyte sedimentation rate), and relevant autoimmune markers can help identify potentially associated conditions and guide further management [7].

### 1.3. Pyoderma Gangrenosum after Breast Surgery (PSPG)

Postsurgical pyoderma gangrenosum (PSPG) is a specific type of pyoderma gangrenosum that develops following breast surgery. A systematic review analyzing published reports from 1946 to 2013 found that the majority of PSPG cases occurred after breast surgery, particularly reduction mammoplasty procedures. Interestingly, over 50% of these cases involved bilateral breast procedures, resulting in bilateral PG. Furthermore, a history of PG, either confirmed or suspected, was only present in 16.8% of the patients, suggesting that PSPG development is not always predictable or linked to previous PG occurrences [8].

### 1.4. Impact and Presentation

While relatively uncommon (approximately three cases per million people per year in the US) [8], PG following breast surgery can significantly impact a patient’s physical and psychological well-being. The initial presentation is often misdiagnosed as a wound infection due to its close resemblance. Typical symptoms include a rapidly expanding, painful ulcer at the surgical site. The ulcer may display undermined edges, violaceous borders, and necrotic tissue. Although extensive, the ulceration tends to be sharply demarcated and spares the nipple-–areola complex. The onset of PG can vary, with some cases developing immediately after surgery, while others may occur weeks or even months later, typically within an average of 7 days after surgery [9]. In terms of underlying factors, PSPG has a different pattern compared to other types of PG. While conditions like inflammatory bowel disease and rheumatoid arthritis are commonly associated with pyoderma gangrenosum, PSPG tends to have a lower association with systemic diseases. Instead, it is most frequently associated with hematologic disorders and malignancies. Numerous studies have demonstrated an increased incidence of PSPG in patients with hematologic malignancies such as leukemia, lymphoma, and myelodysplastic syndromes. The precise mechanisms contributing to this association are not yet fully understood, but it is believed that the immune dysregulation seen in these conditions may play a role in the development of PSPG [3].

### 1.5. Treatment

Managing PG after breast surgery (PSPG) requires a multifaceted approach. Systemic immunosuppressive medications like corticosteroids, cyclosporine, or azathioprine address underlying conditions and suppress the immune response to prevent further tissue destruction [3]. The choice between conservative (medical) or surgical management depends on factors like ulcer size, disease severity, and the risk of pathergy (worsening of lesions after manipulation). In many cases, a combination of both approaches may be necessary. Medical treatment, typically involving immunosuppressive medications, plays a central role. Surgery, particularly debridement (removal of necrotic tissue), skin grafting (replacing damaged tissue), or negative pressure wound therapy (promoting healing and preventing infection), is reserved for extensive or rapidly progressing ulcers due to the risk of pathergy [10]. A qualified plastic surgeon experienced in PSPG is crucial for surgical intervention. Close monitoring and adjustments to the treatment plan are essential throughout the course. While PSPG can be challenging, positive outcomes are achievable with proper medical and surgical management [10]. It is important to note that surgical treatments may require multiple procedures, and medical therapies often work alongside surgery to control the autoimmune response and prevent further ulcer development [11].

The implications of PG in surgical outcomes require a deep understanding of its pathophysiology to optimize patient care and minimize potential complications. This review aims to compare conservative and combined treatment modalities, emphasizing the need for tailored management strategies to handle the unique challenges posed by PG. Furthermore, we present a detailed case report exemplifying the diagnostic and therapeutic challenges posed by PG and providing a real-world context to the theoretical insights of this review.

## 2. Case Report

Complications following breast surgery can pose significant challenges in management, especially in patients with underlying autoimmune conditions. This case report presents the successful management of a patient who developed extensive PG ulcerative lesions following breast surgery. The treatment plan involved a combination of systemic therapy, vacuum-assisted closure (VAC) therapy, and surgical intervention.

### 2.1. Case Presentation

A 48-year-old female patient visited our clinic seeking breast surgery, labiaplasty, and vaginoplasty. She had a history of suspected Crohn’s disease, although it was not confirmed. No other autoimmune diseases were reported. Following consultation, we agreed to perform bilateral auto-augmentation mastopexy, vaginoplasty, and labiaplasty. Preoperative investigations, both clinical and paraclinical, showed normal results. The surgery, conducted under general anesthesia, proceeded without complications. During the labiaplasty, vaginoplasty, and at the STAG donor site, we did not observe any pathergy. The wounds healed rapidly and without complications, even in the setting of immunosuppression in the abdominal donor area. The patient’s postoperative recovery was positive, and she was discharged after two days.

### 2.2. Diagnosis and Treatment

**Figure 1:**
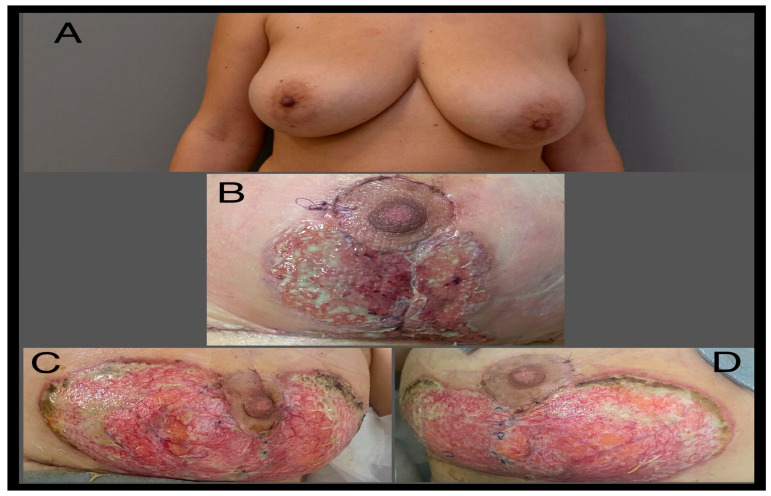
Sequential clinical presentation of PG post-breast surgery. (**A**) Preoperative condition serving as a baseline; (**B**) day 7 post-operation, illustrating initial inflammatory symptoms and wound dehiscence; (**C**,**D**) day 14 post-operation, displaying the progression of PG after surgical debridement and initiation of vacuum-assisted therapy, highlighting the rapid development of the condition.

However, on postoperative day 7 (POD 7), the patient presented with red, firm, and tender breasts, along with areas of wound dehiscence at the lower pole. She developed a fever of 39 °C, accompanied by severe pain and swelling in both breasts. Hospital admission was necessary due to the rapid progression of ulcerative lesions in the bilateral lower pole of the breasts. Despite antibiotic treatment, the ulcerative lesions continued to progress rapidly, reaching dimensions of 17.5 × 10 cm on each breast. Laboratory analyses revealed anemia (Hb = 8.3 g/dL), leukocytosis (leukocytes = 26,610), and elevated CRP levels (28.58 mg/dL) (Figure 1).

**Figure 2:**
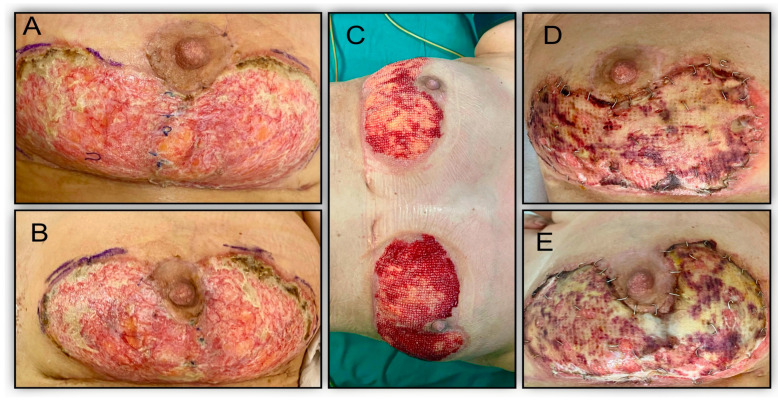
Progressive stages of PG management over a six-week period. (**A**,**B**) Lesions undergoing conservative treatment including corticosteroids, topical tacrolimus, moist dressings, and hyperbaric oxygen therapy, demonstrating no significant improvement, indicating a persistent, non-resolving progression. (**C**) The condition of the lesions after 3 weeks of preparatory vacuum therapy, showing well-formed granulation tissue in readiness for skin grafting. (**D**,**E**) The affected area post-skin grafting with vacuum therapy still in application to promote graft take and wound healing. (**E**) The surgical site one week after vacuum therapy was discontinued, presenting the initial healing stages post-grafting.

Surgical intervention was performed on POD 8, involving surgical debridement and the application of vacuum therapy (with negative wound culture results). On POD 11, the vacuum therapy kit was changed, and it was subsequently discontinued on POD 14 due to the progression of ulcerative lesions under VAC therapy. Based on negative cultures, the characteristic features of the lesions, and a dermatological consultation, the suspected diagnosis was pyoderma gangrenosum. Conservative treatment, including local dressing with silver sulfadiazine, systemic antibiotic therapy, and corticosteroids (dexamethasone 1 mg/kg), was initiated. Laboratory analyses showed a favorable progression, with stabilization and no further progression of the ulcerative lesions upon discharge on POD 20 (leukocyte count 13,990, hemoglobin 10.6 g/dL, CRP 0.64 mg/dL). However, after 6 weeks of continued conservative treatment with additional modalities like hyperbaric therapy, moist dressings (Hydroclean Advance), and topical tacrolimus, the wounds showed no further improvement. Consequently, a decision was made to proceed with skin grafting. Vacuum therapy was initiated alongside systemic corticosteroid therapy to promote lesion granulation and prepare for grafting. The vacuum kit was changed after 5 days, resulting in favorable progress. After 3 weeks of vacuum therapy, positive advancements were observed with a granular lesion appearance and absence of necrotic residue, although local lesion spread remained high (Figure 2).

In the 11th week following the initial surgery, we opted to address the ulcerative lesions through full-thickness skin grafting sourced from the abdomen. Subsequently, an abdominoplasty was performed, and the skin was deepidermized. Primary closure of the abdomen was achieved, and the ulcerative lesions underwent skin grafting after minimal debridement. Vacuum therapy was administered to the skin grafts at -75 mmHg. After 5 days, vacuum therapy was discontinued, and progress was favorable. The skin grafts successfully integrated, and we proceeded with a Grassolind gauze. Within 2 weeks, the skin grafts were fully integrated. The decision to proceed with a full-thickness skin graft from the abdomen three weeks after the diagnosis was made due to the extensive size of the lesions and the lack of response to conservative treatment. This approach allowed for the combination of an aesthetic procedure (abdominoplasty) with the benefit of avoiding additional scars that would have resulted from harvesting split-thickness grafts from the thigh. Although the decision for surgical intervention was made early on, the actual grafting was performed five weeks later, following vacuum therapy to optimize the wound bed. 

**Figure 3:**
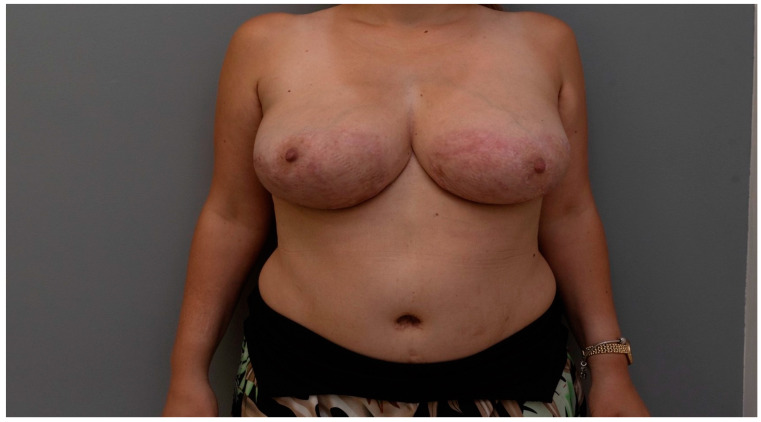
The outcome of surgical therapy for PG with skin grafting after 5 months is presented. Although visible scars are apparent, it is anticipated that within 6–7 months, the aesthetic appearance of the scars will improve significantly.

While the specific long-term monitoring plan for this patient was not explicitly detailed in this case report, we acknowledge the importance of ongoing surveillance in PG patients. Following the principles outlined in the general PG management guidelines of the American Academy of Dermatology (AAD) and the European Dermatology Forum (EDF), we recommend a multidisciplinary approach to long-term care. This would include regular immunologic testing, such as complete blood counts to monitor for anemia and systemic inflammation, autoantibody panels (ANA and RF) to screen for potential autoimmune conditions, and inflammatory markers like C-reactive protein and erythrocyte sedimentation rate. Given this patient’s history of gastrointestinal issues and suspected Crohn’s disease, continued monitoring by a gastroenterologist is also crucial. If the patient experiences recurrent gastrointestinal symptoms like abdominal pain, rectal bleeding, or diarrhea, a colonoscopy may be necessary to rule out inflammatory bowel disease (Figure 3).

We further aimed to provide a comprehensive overview of available treatment options for PG. We examined conventional approaches, emerging therapies, multidisciplinary interventions, and surgical interventions to equip healthcare professionals, researchers, and patients with valuable insights into managing this challenging condition.

## 3. Literature Search

A systematic literature review was conducted to identify relevant studies that discuss the management strategies for PG following breast surgery, emphasizing the comparison of conservative and combined treatment modalities. The review aimed to cover publications from 1 January 2018 to 31 December 2023, reflecting the most recent advancements and perspectives in the field.

### 3.1. Search Strategy

A comprehensive search was performed using the PubMed database. The search strategy combined MeSH terms and keywords, including “pyoderma gangrenosum”, “breast”, “breast surgery”, “treatment”, and “surgical”. Given the rarity of PG, all case reports, case series, and clinical trials focusing on PG in association with breast surgery were included. Reviews and meta-analyses (secondary studies) and articles that were not available in English were excluded. The initial search retrieved 35 results. After applying the inclusion and exclusion criteria, 24 articles were considered relevant for further analysis. The excluded articles typically focused on PG in different body regions or discussed primary diseases with PG as a secondary complication. We aimed to avoid confusion and biases or attributing false morbidity to this condition.

### 3.2. Data Extraction

The studies were subsequently reviewed and divided into categories based on the management strategies they described: conservative medical management alone or surgical intervention in conjunction with medical therapy. This organization allows for a comparative analysis of the management outcomes, assessing both the healing process and the rate of complications. In addition, to grade the results of the articles, abbreviations and scores were used to describe every article easily. A score of 0 indicated no improvement or disease progression despite treatment, and a score of 1 represented disease remission, which included complete healing of the ulcers. The abbreviation S was used for surgical treatment, and the abbreviation C for conservative treatment. The chronological order of these letters reflects the sequence of treatment modalities applied. For example, ‘S0, C1’ implies that initial surgical intervention did not yield a positive outcome, whereas subsequent conservative management resulted in remission.

## 4. Results

Our systematic search of PubMed identified 24 relevant articles published between 2018 and 2023 that explored treatment options for PG following breast surgery. These studies investigated conservative (n = 15) and combined treatment strategies (n = 9). Conservative management, relying on medications like corticosteroids to control inflammation, achieved complete remission in most studies (13 out of 15 studies). However, complications like flap loss were reported in two studies [12]. Combined management, incorporating both conservative therapy and surgical interventions like debridement or grafting, was more heterogeneous. While two studies showed remission with this approach, the relative effectiveness of surgery versus medication remained unclear [13,14]. Interestingly, four studies highlighted the potential benefits of surgical approaches, including debridement, grafting, flaps, and negative pressure therapy, with varying success rates [15,16,17,18]. Conversely, three studies discouraged surgery due to the risk of worsening lesions and additional complications [19,20,21]. The detailed outcomes and summaries of each article are provided in Table 1.

## 5. Discussion

Postsurgical pyoderma gangrenosum is a complex, autoinflammatory condition that frequently complicates recovery after surgical procedures, particularly following breast surgery. This investigation aimed to advance our understanding of the efficacy and safety of surgical interventions in PG management and to offer insights into a spectrum of therapeutic options for this complex condition.

### 5.1. The Importance of Early Diagnosis

Early diagnosis and intervention are critical in managing PSPG, as delayed treatment can lead to severe complications, including extensive tissue damage and unnecessary surgical interventions [17,22,23,26,27]. Studies consistently highlight the importance of distinguishing PSPG from other postoperative complications like infections, which often look similar but require drastically different treatment approaches [23,26]. As demonstrated in our case, where a 48-year-old female developed extensive PG lesions following breast surgery, the rapid progression from initial symptoms to severe ulcerative conditions highlights the necessity for vigilance and prompt action.

### 5.2. Effectiveness of Management Strategies

The consensus across studies favors medical management, primarily immunosuppression, over surgical interventions. Immunosuppressive therapies, including corticosteroids and cyclosporine, have been pivotal in managing PSPG, as highlighted in cases from multiple studies [16,24,28,34]. However, the timing of the transition from aggressive wound care to surgical closure, including skin grafting, remains a critical decision point. While corticosteroids can effectively suppress inflammation, their prolonged use at high doses is associated with significant adverse effects, including immunosuppression, impaired wound healing, increased susceptibility to infections, and poor integration of skin grafts [36]. Our experience supports the safety profile of surgical procedures, with no adverse events related to the surgical technique itself observed in our case report. However, although surgical interventions can be safe, they must be approached with caution due to the complexity and high risk of exacerbating the condition. The trauma inflicted during the procedure can trigger the development of new lesions at the surgical site or exacerbate existing ones, a phenomenon known as pathergy. The formation of pathergy lesions complicates the surgical management of PG and underscores the challenges in achieving long-term disease control through surgical means alone. This aligns with the broader literature, which advocates conservative management as a first-line approach, with surgery reserved for specific, well-justified cases [1,2].

Innovative treatments such as negative pressure wound therapy (NPWT) and hyperbaric oxygen therapy have also been explored. NPWT, for instance, is used to prepare and stabilize wound beds before attempting more invasive procedures like skin grafting, which has been shown to improve outcomes in difficult cases [20]. Similarly, hyperbaric oxygen therapy has been documented to accelerate healing by enhancing oxygen delivery to hypoxic tissue, which is crucial in severe cases of PSPG where rapid wound closure is desired [17,23].

### 5.3. Challenges in Management

The differential diagnosis of PSPG is faced with challenges due to its rarity and the lack of specific diagnostic markers. Misdiagnosis leads to inappropriate treatments that can aggravate the condition. Moreover, managing PSPG in patients with systemic diseases such as cancer or autoimmune disorders complicates therapy due to the systemic effects of immunosuppressive drugs and the delicate balance needed to manage both conditions [16]. The variability in the clinical presentation of PG and the response to treatment requires customized therapeutic strategies. Our review of 24 articles published between 2018 and 2023 revealed a preference for conservative corticosteroid treatment, while showcasing the potential benefits of surgical interventions in selected cases. The decision-making process, therefore, must consider the specific characteristics of each case, including the severity of the lesions, the presence of pathergy, and the patient’s overall health and concurrent conditions.

It is essential to acknowledge the limitations of our study. The scarcity of case studies on surgical treatment for PG makes it challenging to comprehensively contextualize our findings within the existing literature. This limited pool of comparable data hinders our ability to conduct robust comparative analyses and draw definitive conclusions regarding the overall efficacy and safety of surgical interventions for PG management. These limitations highlight the need for larger, long-term studies to further validate our findings and elucidate the optimal management strategies for PG.

There remains a substantial gap in the literature regarding standardized diagnostic criteria and management protocols for PSPG. Future research should focus on developing diagnostic biomarkers and more effective, less harmful treatment modalities. Additionally, understanding the pathophysiology of PSPG could unveil new therapeutic targets, potentially reducing the reliance on broad immunosuppression and its associated risks. Ensuring rigorous postoperative care and ongoing monitoring is critical to addressing complications promptly and adjusting treatment as the patient’s condition evolves. This phase emphasizes the importance of continued collaboration among specialists, including infectious disease consultants, when infection is a concern in providing comprehensive care. Regular follow-ups and wound care, tailored to minimize pathergy risks and adapted based on healing progress, are essential components of this collaborative care model. The dynamic nature of the understanding of PG, especially in post-operative contexts, highlights an ongoing need for interdisciplinary research and dialogue.

## 6. Conclusions

Our study suggests that surgical interventions, implemented under appropriate immunosuppressive regimens, may be a safe and potentially beneficial approach for managing cases of PG. While our findings demonstrate promising results regarding wound healing and aesthetic outcomes, the limited scope of the study necessitates further investigation. Future research efforts, particularly larger, controlled trials, are crucial to validate these preliminary observations and definitively establish the role of surgery in PG treatment protocols.

## Figures and Tables

**Table 1:** Outcomes of conservative and surgical treatments for PSPG.

Study	Treatment Type	Case Summary	Score
Solis et al. [22]	Conservative	Complete resolution after 3 months of corticotherapy.	C1
Guaitoli et al. [23]	Conservative	Complete healing after 6 months of corticotherapy and hyperbaric oxygen therapy; steroids phased out.	C1
Song et al. [24]	Conservative	Healing achieved after 6 months of corticotherapy.	C1
Rich et al. [25]	Conservative	Wounds improved with secondary healing using corticosteroids, infliximab, and mycophenolic acid. Surgery not advised due to COVID-19 risks.	C1
Hammond et al. [26]	Conservative	Complete wound closure within 51 days using corticotherapy (4 cases analyzed).	C1
Cabanas et al. [27]	Conservative	Complete healing after 2 months of corticotherapy and cyclosporine A.	C1
Tomoda et al. [28]	Conservative	Initial improvement with corticotherapy and infliximab; worsened after 1 month due to ulcerative colitis exacerbation.	C0, C1
Li et al. [12]	Conservative	Of 8 patients, 4 responded well to corticotherapy without complications; 4 experienced complications, including flap loss and acute lung injury.	C0, C1
Nicksic et al. [29]	Conservative	Successful treatment of 87 patients using steroids; surgery not recommended.	C1
Zapata et al. [30]	Conservative	PG resolved appropriately after starting corticotherapy.	C1
Ariane et al. [31]	Conservative	Complete remission of PG wound after 7 months of corticotherapy.	C1
Ljubojević et al. [32]	Conservative	A 45-year-old man with multiple ulcers improved partially with corticotherapy and sulfasalazine.	C1
Coste et al. [33]	Conservative	A 38-year-old woman’s ulcerative lesions post-breast surgery were resolved with systemic steroid treatment after failure of antibiotics.	C1
Ramamurthi et al. [19]	Conservative, Surgical	Postsurgical PG after DIEP breast reconstruction treated initially with prednisone, then infliximab. Surgical debridement and delayed skin grafting needed due to complications.	S0, C1
Kim et al. [13]	Conservative, Surgical	Postsurgical PG after inferior sectorectomy managed with prednisone and topical steroids, followed by surgical debridement and vacuum-assisted closure.	C1, S1
Mella et al. [15]	Conservative, Surgical	Four cases analyzed; all involved prednisone treatment and surgical interventions, including debridement and skin grafting due to non-healing wounds.	C0, S1
Vernaci et al. [16]	Conservative, Surgical	Subclavicular CVC insertion complications managed with corticotherapy, followed by surgical debridement and skin grafting. Further surgery needed for PG complications.	C0, S1
Hirai et al. [20]	Conservative, Surgical	Bilateral mastectomy complications managed with immediate surgical debridement and subsequent prednisone treatment. Skin grafting was eventually required.	S0, C1
Rogers et al. [21]	Conservative, Surgical	Multidisciplinary management of PG after excisional biopsy of the breast required multiple surgeries and corticotherapy for resolution.	S0, C1
Canzoneri et al. [14]	Conservative, Surgical	PG after body-contouring surgery managed with a month of corticotherapy and subsequent breast reconstruction without further complications.	C1, S1
Costa et al. [17]	Conservative, Surgical	Postsurgical complications from breast conservation surgery managed with surgical debridement and immunosuppressive therapy.	C0, S1
Mullholand et al. [18]	Conservative, Surgical	Patient with diabetes developed PSPG post-breast cancer surgery; managed with non-steroidal treatments due to diabetes.	C0, S1
Figuerelli et al. [34]	Conservative, Surgical	Patient developed PSPG post-breast surgery; managed with hyperbaric oxygen and surgical debridement, followed by immunosuppressive therapy.	C1, S0
Edinger et al. [35]	Conservative, Surgical	Four patients with PSPG managed with delayed diagnosis and treatment using prednisone and sometimes cyclosporin, followed by surgical interventions.	C1, S0

PG: pyoderma gangrenosum; PSPG: postsurgical pyoderma gangrenosum; CVC: Central Venous Catheter; DIEP: Deep Inferior Epigastric Perforator (type of breast reconstruction).

## Data Availability

Data sharing is not applicable to this review as no new data were generated.
